# Hypotension in ICU Patients Receiving Vasopressor Therapy

**DOI:** 10.1038/s41598-017-08137-0

**Published:** 2017-08-17

**Authors:** Bryce Yapps, Sungtae Shin, Ramin Bighamian, Jill Thorsen, Colleen Arsenault, Sadeq A. Quraishi, Jin-Oh Hahn, Andrew T. Reisner

**Affiliations:** 1 Department of Mechanical Engineering, University of Maryland, College Park, MD 20742 USA; 2 Department of Emergency Medicine, Massachusetts General Hospital, Boston, MA 02114 USA; 3 Department of Anesthesia, Critical Care and Pain Medicine, Massachusetts General Hospital, and Harvard Medical School, Boston, MA 02114 USA

## Abstract

Vasopressor infusion (VPI) is used to treat hypotension in an ICU. We studied compliance with blood pressure (BP) goals during VPI and whether a statistical model might be efficacious for advance warning of impending hypotension, compared with a basic hypotension threshold alert. Retrospective data were obtained from a public database. Studying adult ICU patients receiving VPI at submaximal dosages, we analyzed characteristics of sustained hypotension episodes (>15 min) and then developed a logistic regression model to predict hypotension episodes using input features related to BP trends. The model was then validated with prospective data. In the retrospective dataset, 102-of-215 ICU stays experienced >1 hypotension episode (median of 2.5 episodes per day in this subgroup). When trained with 75% of retrospective dataset, testing with the remaining 25% of the dataset showed that the model and the threshold alert detected 99.6% and 100% of the episodes, respectively, with median advance forecast times (AFT) of 12 and 0 min. In a second, prospective dataset, the model detected 100% of 26 episodes with a median AFT of 22 min. In conclusion, episodes of hypotension were common during VPI in the ICU. A logistic regression model using BP temporal trend features predicted the episodes before their onset.

## Introduction

Many patients treated for circulatory shock receive hours or days of vasopressor infusion to manage persistent hypotension. Vasopressors generally act by elevating vascular tone, cardiac contractility and heart rate, and these physiological effects result in higher arterial blood pressure (BP). Administration of vasopressors to prevent mean arterial pressure (MAP) < 65 mmHg for sepsis is recommended by the consensus Surviving Sepsis guidelines^1^, while Advanced Cardiac Life Support (ACLS) advises vasopressor therapy for systolic BP < 70 mmHg^2^. The rationale for treating severe hypotension with vasopressors is based in large part on classical physiology studies showing that central nervous system (CNS) auto-regulation of CNS perfusion fails in healthy animals for MAP < 65 mmHg^3, 4^. Below this limit, CNS hypoperfusion and tissue ischemia occur. Indeed, associations between duration of hypotension and measures of end-organ injury have been reported in critically-ill^5–7^ and intraoperative^8^ patient populations.

Vasopressor infusion requires ongoing titration of dose to ensure that the patient’s MAP is high enough to prevent hypotension, but not too high, which can result in unnecessary cardiac demand, cardiac ectopy, and excessive vasoconstriction that impairs tissue perfusion. As a practical matter, the infusion dose must be continually re-adjusted through time, because the critically ill patient’s underlying condition is not static, leading to either an increase or decrease in the dose necessary for maintaining a target MAP.

Recent capabilities in electronic data archiving for the ICU reveal that rates of compliance with BP goals may be suboptimal. In a preliminary study^9^, we reported that ICU patients frequently experience sustained episodes (>15 min) of hypotension while receiving vasopressor infusions. In a related report, Hawryluk *et al*. found that nearly half of BP measurements in ICU patients with acute spinal cord injuries were below the BP range that is recommended by consensus guidelines to prevent secondary injury to newly damaged spinal cord^10^. Therefore, the first goal of this investigation was to better understand compliance with BP management guidelines during ICU vasopressor administration.

The second goal of this study was to investigate the efficacy of statistical methods for predicting when the patient’s vasopressor dosage requires a change, because computerized decision-support offers a potential means to enhance clinician practice. We sought to develop and evaluate a simple statistical model that might be applied in an ICU as a tool for preventing hypotension by providing a reliable advance warning before hypotension develops.

Overall, if compliance with BP management guidelines during vasopressor infusion is suboptimal, that suggests that ICU patients may be suffering preventable harm. For example, failure to maintain MAP > 65 mmHg via suitable vasopressor infusion doses might be a causal factor (via CNS ischemia) to the permanent cognitive impairment reported in many survivors of septic shock^11^ or suboptimal outcomes after acute spinal cord injury^10^. On the other hand, if standard statistical methods are efficacious at predicting when dose changes are necessary, even before hypotension develops, then statistical models might offer a relatively practical method for improving compliance rates with BP targets and avoiding preventable end-organ injury during vasopressor therapy.

## Methods

### Setting

We studied an archived dataset of adult patients from a mixture of ICUs from a single institutional hospital (“Hospital 1”). Prospectively, we collected a second dataset from the surgical ICU of our institution (“Hospital 2”) to rigorously validate our statistical model.

### Subject selection

We studied ICU patients with vasopressor infusion doses documented in the nursing flowcharts (dopamine, epinephrine, norepinephrine, or phenylephrine) with concurrent heart rate (HR) and MAP data from indwelling arterial catheters. Only records from patients who survived at least 48 hours after their ICU stays were included in this study, to exclude patients who may have been comfort measures only (CMO) and therefore not receiving aggressive BP management.

### Data collection

For the Hospital 1 dataset, the MIMIC II database (MIMIC II, the Multi-Parameter Intelligent Monitoring in Intensive Care II Database, version 3) was accessed through the PhysioNet website^12^. Minute-by-minute numeric data of MAP and HR originally sourced from the bedside monitor, as well as vasopressor dose information and MAP charted in the nursing documentation, were downloaded for analysis.

For the Hospital 2 dataset, we prospectively identified patients in the surgical ICU receiving vasopressor infusions. MAP and HR were collected by the BedMaster data archiving system (Excel Medical, Jupiter, FL) while vasopressor dose information and intermittent MAP measurements were obtained by chart review of nursing documentation.

### Data pre-processing

For both datasets, a moving “causal” 5 min median filter was applied to the MAP data (where the filtered value of MAP was determined as the median of the preceding 5 min data). We assumed vasopressor was infused no more than 60 min after its last documented infusion.

We focused our study of dose-titration behavior of clinicians on specific data intervals. Specifically, within each patient record, data intervals were excluded from primary analysis when the interval (a) lacked either MAP or vasopressor dose data, (b) contained non-physiological MAP measurements (<5 mmHg or >300 mmHg), (c) occurred during the first 30 min of a patient’s ICU stay, when initial resuscitation was occurring, (d) occurred during maximum vasopressor infusion defined as per hospital protocols, or (e) contained two or more simultaneous vasopressor infusions. This left us with paired dose and MAP data during vasopressor mono-therapy at sub-maximum infusion doses. This dataset was deemed optimal for studying the dose-titration behavior of clinicians.

The validity of the recorded MAP was confirmed by comparing it against contemporaneous non-invasive MAP values, from the nursing documentation.

We also performed a secondary analysis by relaxing the exclusion criteria (c), (d), and (e), for assessing the performance of the statistical model under wider-ranging conditions.

### Study outcome

The study outcome was sustained hypotension. For each patient record, hypotensive measurements were identified (MAP < 60 mmHg) and hypertensive measurements were also identified (MAP > 100 mmHg). Next, sustained episodes of hypotension were identified, defined as 15 min or more of continuous hypotension. Episodes begin upon the first hypotensive MAP sample and terminate with any subsequent in-range MAP sample, defined as 100 mmHg ≥ MAP ≥ 60 mmHg.

### Characteristics of sustained episodes of hypotension

Patients’ ICU stays were divided into three categories: ICU stays with sustained hypotension, ICU stays with only transient (non-sustained) hypotension (<15 min), and ICU stays without any hypotension. For each category, we computed: demographic statistics of patients receiving at least 30 min of vasopressor infusion; documented reason(s) for vasopressor infusion based on the patient’s discharge summary; and descriptive statistics about vasopressor infusion and MAP trends during vasopressor infusion.

For the ICU stays with at least one sustained episode of hypotension, we analyzed vasopressor dose changes before and after the onset of the episodes, to characterize clinician behavior before and after the onset of the episodes. Specifically, we analyzed the incidence of dose changes in vasopressors within 30 min prior to the onset of hypotension and after the onset of hypotension. For the Hospital 2 dataset, we also examined whether the episodes of hypotension were associated with additional doses of sedatives; other cardiovascular medications; or documented clinical event within the nursing note.

Many episodes of hypotension were followed within 30 min by a subsequent episode of hypotension. Sequential episodes of sustained hypotension separated by 30 min or less were termed “episode spans.”

### Logistic regression model for predicting sustained hypotension

We developed a logistic regression model for predicting sustained episodes of hypotension, using cases that account for 75% of the data from the Hospital 1 dataset. We performed analysis in the MATLAB computing environment (Mathworks, Natick MA), using the greedy backward feature selection until only statistically significant features remained (P < 0.05 significance level).

We explored 54 candidate predictor features derived from the MAP trend, vasopressor trend, and HR trend data: mean, least-squares slope, and standard deviation of MAP, vasopressor dosage, and HR over time-scales of 5, 10, 20, 30, 45, and 60 min. These are features that are available at the bedside via electronic interfaces with the patient monitor and the infusion pump and do not require additional clinical information. We computed these features at 5 min time steps throughout the duration of vasopressor infusion of each subject. We normalized the candidate features before application to logistic regression.

There were a total of 54,276 time point observations used for training the model, after excluding time points that lacked a complete set of feature data. For each time point, we calculated whether or not it was followed by sustained hypotension within the subsequent 15 min and used it as the model output for logistic regression. We used the MATLAB routine *glmfit* to develop the logistic regression model.

In addition to the above primary analysis, we also performed a secondary analysis to test whether the aforementioned primary logistic regression model was improved with additional candidate predictor features, adding parameters about the individual patient’s age, gender, vasopressor name, and type of shock pathology, i.e., presence of sepsis (yes/no), presence of hemorrhagic hypovolemia (yes/no), etc., using the chart review methodology described above.

### Logistic regression model retrospective evaluation

The first evaluation metric was model goodness-of-fit. For deciles of the dataset, we compared the model’s predicted versus the actual probabilities that any time point would be followed within 15 min by sustained hypotension. We did not apply a formal goodness-of-fit test, i.e., Hosmer-Lemeshow test, because it is not reliable for datasets larger than 50 K samples (the formal test becomes 100% sensitive to trivial deviations from ideal fit given large datasets^13^).

Our second set of evaluation metrics was the trade-off between sensitivity and specificity given different thresholds for the model output. Because the model output at any given time *t* was very similar to the model output one minute later, we studied alarm episodes rather than minute-by-minute output. An alarm episode was defined as the continuous time interval when the logistic regression output exceeded the alarm threshold. An alarm episode was a “false alarm” if, directly upon its onset, there was no sustained hypotension commencing within 30 min. An episode of hypotension was “detected” if, directly upon its onset, there had been an alarm episode in the preceding 30 min. We computed the following statistics through a range of logistic regression output thresholds for alarming: false alarms per 24 hours; undetected episodes of hypotension; and advance forecast time prior to the onset of the episode of hypotension.

Finally, we compared the logistic regression model with two simple threshold alerts that alarmed any time that MAP < 60 mmHg or MAP < 65 mmHg, respectively, representing default functionality that can be achieved with today’s bedside patient monitors. For these comparisons, we fixed the alarm threshold of the logistic regression model output to match the sensitivity of the simple MAP < 60 mmHg threshold alert: that simple threshold alert offered a sensitivity of 100% for detecting hypotension, therefore, we selected the threshold for the logistic regression model output that offered 100% sensitivity in the Hospital 1 training dataset.

The logistic regression model and the simple threshold alerts were then evaluated using the Hospital 1 testing dataset (25% of the overall Hospital 1 dataset). We computed the following statistics: false alarms per 24 hours; undetected episodes of hypotension; advance forecast time prior to the onset of the episode of hypotension; and duration of the alarm episodes. These metrics were compared using the Wilcoxon Rank Sum test.

### Logistic regression model prospective evaluation

After collecting the *de novo* prospective dataset collected from Hospital 2, we repeated the comparison between the logistic regression model (using the same threshold used in the retrospective evaluation) and simple threshold alerts. We repeated the same evaluation methodology with the goal of evaluating the generalizability of the results from Hospital 1, i.e., whether the logistic regression model was externally valid outside of Hospital 1. We computed the same performance metrics, and again compared the alerting methods using the Wilcoxon Rank Sum test.

### Comparing documented dose increases versus logistic regression model output

We sought to explore the relationship between documented dose increases versus the logistic regression model output. We identified instances where the vasopressor dose was documented to increase ≥50%, and we calculated the proportion for which the logistic regression model was predicting sustained hypotension.

## Results

### Characteristics of sustained episodes of hypotension

A total of 175 patients accounting for 215 ICU stays from Hospital 1 were identified as patients receiving at least 30 minutes of vasopressor mono-therapy at sub-maximal doses. There were 102 ICU stays (47%) with at least one sustained episode of hypotension during vasopressor infusion. These populations are characterized in Tables 1 and 2.

**Table 1:** Characteristics of intensive care unit stays.

	Hospital 1^a^				Hospital 2^a^
	Stays w/sustained hypotension^b^	Stays w/non-sustained hypotension^b^	Stays w/o hypotension^b^	All Stays	All Stays
**Demographics:**					
ICU stays, n (%)	**102** (47)	**57** (27)	**56** (26)	**215** (100)	**62** (100)
Unique patients^c^, n (%)	**90** (51)	**53** (30)	**54** (31)	**175** (100)	**62** (100)
Age, median (IQR)	**75** (64–83)	**74** (63–82)	**70** (59–78)	**74** (64–82)	**69** (61–78)
Female, proportion, %	**45**	**45**	**50**	**49**	**55**
Male, proportion, %	**52**	**51**	**50**	**49**	**45**
Undocumented gender, proportion, %	**3**	**4**	**0**	**2**	**0**
**Indication for vasopressors (subjects may have more than one documented indication):**					
Sepsis or possible sepsis, proportion, %	**47**	**42**	**46**	**48**	**45**
Cardiogenic or possible cardiogenic, proportion, %	**47**	**58**	**54**	**54**	**45**
Post-operative care, proportion, %	**14**	**13**	**22**	**18**	**68**
Other or unknown, proportion, %	**22**	**28**	**43**	**31**	**19**
**Characteristics of vasopressor infusion:**					
Total duration of vasopressor infusion, median per stay (IQR), hr	**21.3** (9.52–46.6)	**17.1** (6.0–37.6)	**5.9** (1.7–11.3)	**14.0** (4.5–34.9)	**37.6** (22.3–55.6)
Time between vasopressor dose changes, median per stay (IQR), min	**90** (55–143)	**90** (36.3–242.5)	**60** (25–90)	**75** (45–138)	**60** (28–110)

^a^Hospital 1 includes patient data from the MIMIC II database and Hospital 2 includes patient data from a separate medical center. ^b^Hypotension is defined as MAP < 60 mmHg and sustained episode of hypotension is defined as at least 15 continuous min of hypotension. ^c^Some patients have multiple stays that are in different categories, therefore the number of unique patients for all stays is less than the sum of the first three columns.

**Table 2:** Characteristics of MAP during mono-vasopressor infusion.

	Hospital 1^a^				Hospital 2^a^
	Stays w/sustained hypotension^b^	Stays w/non-sustained hypotension^b^	Stays w/o hypotension^b^	All Stays	All Stays
**Statistics:**					
MAP during infusion, median per stay (IQR), mmHg	**68** (65–72)	**75** (71–78)	**80** (76–87)	**73** (67–79)	**75** (71–79)
MAP hourly standard deviation during infusion, median per stay (IQR), mmHg	**4.0** (2.9–5.5)	**3.6** (2.3–4.5)	**3.3** (2.5–4.8)	**3.8** (2.7–5.5)	**3.8** (2.9–4.9)
Proportion of 100 ≥ MAP ≥ 60 mmHg, median per stay (IQR), %	**80** (70–88)	**97** (93–99)	**100** (89–100)	**91** (78–98)	**98** (92–99)
Proportion of MAP during transient hypotension, median per stay (IQR), %	**3.7** (1.8–5.9)	**1.3** (0.5–3.5)	n/a	**1.6** (0.0–4.4)	**0.3** (0.0–1.3)
Proportion of MAP during sustained hypotension, median per stay (IQR), %	**11** (4–21)	n/a	n/a	**0.1** (0.0–11)	**0.0** (0.0–2.5)
Proportion of MAP during hypertension^c^, median per stay (IQR), %	**0.1** (0.0–3.0)	**0.1** (0.0–2.9)	**0.2** (0.0–11)	**0.1** (0.0–3.5)	**1.0** (0.0–3.2)
Sustained episodes of hypotension per 24 hours, median per 24 hours (IQR), n	**2.5** (1.1–5.0)	n/a	n/a	**0.0** (0.0–3.0)	**0.0** (0.0–1.2)

^a^Hospital 1 includes patient data from the MIMIC II database and Hospital 2 includes patient data from a separate medical center. ^b^Hypotension is defined as MAP < 60 mmHg and sustained episode of hypotension is defined as ≥15 min; see text for details. ^c^Hypertension defined as MAP > 100 mmHg.

The majority of the episodes of hypotension appeared valid, as we found that only 5.4% of these episodes had two or more MAP measurement samples that differed by more than 10 mmHg from the documented non-invasive MAP measurements. During episodes, the difference between MAP measurements and non-invasive MAP measurements was 5.1 ± 6.6 mmHg (mean ± std. dev.).

Compared to ICU stays without hypotension, ICU stays with sustained episodes of hypotension had significantly longer durations of vasopressor infusion, lower MAP during vasopressor infusion, and lower overall proportion of in-range MAP (P < 0.05 per Mann-Whitney U and Chi-squared tests).

There were no additional significant differences in the characteristics in Table 1 and Table 2, including no significant difference in terms of the clinical indication for vasopressor infusion therapy (based on co-review of 20 records to determine one or more clinical indications, Cohen’s κ was 0.79).

### Sustained episodes of hypotension: clinical responses

**Figure 1:**
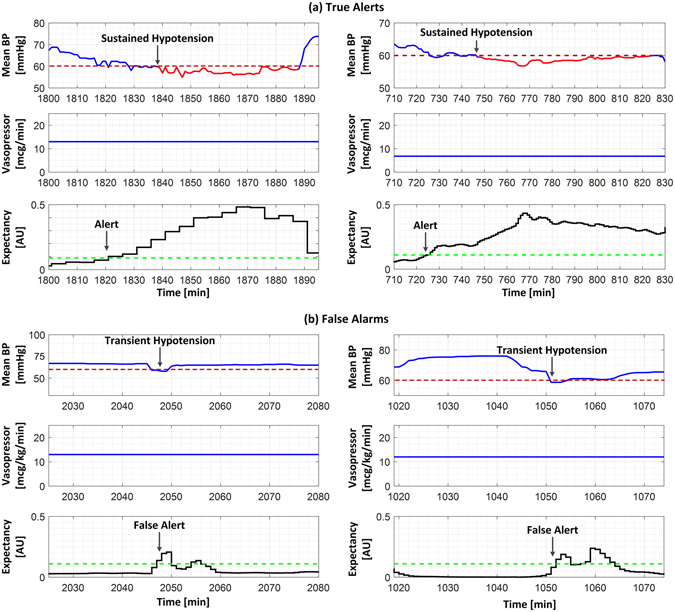
Examples of the investigational logistic regression model detecting sustained episode of hypotension (top panels) and triggering false alerts (bottom panels). At each time point, the logistic regression model used MAP measurements of the previous 60 min to compute an “expectancy” value that an episode of hypotension will be occurring within 15 min. Top panels: Two typical examples of successful prediction of sustained hypotension, in which sustained hypotension was preceded by steady decreases in the patient’s MAP, without any adjustments in vasopressor dose. In these examples, the model’s output value passed above the output threshold for alerting 17 (left) and 21 (right) minutes prior to the onset of the sustained episode of hypotension. Bottom panels: Two typical examples of false alerts occurring in the setting of transient hypotension that quickly resolved without any intervention.

In Hospital 1, there were 354 episode spans (defined as sequential episodes of hypotension separated by 30 min or less). Most (92%) episode spans resolved without *any* documented dose increase in vasopressors. The top two panels in Fig. 1 show representative examples of an episode of hypotension without any adjustment of the vasopressor dose. It was the minority of episode spans that resolved in temporal proximity to a documented increase in vasopressor dose, and these were significantly briefer in duration (P < 0.05), and typically only required a single dose increase. See Table 3.

**Table 3:** Vasopressor dosing before and during sustained episodes of hypotension.

	Hospital 1^a^		Hospital 2^a^	
	Episode resolved^b^ *w/* increase of vasopressor dose	Episode resolved^b^ *w/o* increase of vasopressor dose	Episode resolved^b^ *w/* increase of vasopressor dose	Episode resolved^b^ *w/o* increase of vasopressor dose
**Statistics:**				
Episodes, n (%)	**49** (8)	**562** (92)	**8** (31)	**18** (69)
Episode spans^c^, n (%)	**28** (8)	**326** (92)	**6** (33)	**12** (67)
Duration of episode spans^c^, median (IQR), min	**29** (23–44)	**38** (21–72)	**27** (23–30)	**24** (16–111)
Proportion of MAP < 60 mmHg during episode span^b^, median (IQR), %	**100** (92–100)	**100** (89–100)	**100** (100–100)	**100** (90–100)
**Vasopressor dose changes preceding episode onset:**				
Episode span^c^ onsets with no preceding dose change, proportion, %	**61**	**83**	**83**	**33**
Episode span^c^ onsets with preceding dose decrease, proportion, %	**25**	**6.1**	**17**	**25**
Episode span^c^ onsets with preceding dose increase, proportion, %	**14**	**11.0**	**0**	**42**
**Vasopressor dose changes during episode:**				
Episode spans^c^ with at least one dose increase, proportion, %	**100**	**11**	**100**	**17**
Episode spans^c^ resolved^b^ with a single dose increase, proportion, %	**86**	**0**	**83**	**0**
Total number of dose increases during episode span^c^, median (IQR), n	**1** (0–3)	**0** (0–1)	**1** (1–2)	**0** (0–1)
Time until first dose increase, median (IQR), min	**22** (8–35)	**n/a** ^**d**^ (40–n/a^d^)	**15** (10–22)	**n/a** ^**d**^ (73–n/a^d^)

^a^Hospital 1 includes patient data from the MIMIC II database and Hospital 2 includes patient data from a separate medical center. ^b^Episodes of hypotension were categorized as “resolved with increase of vasopressor dose” if there was a vasopressor dose increase within 30 min of the resolution of the episode. ^c^A sustained (>15 min) episode of hypotension that followed within 30 min another sustained hypotensive episode was regarded as a continuation of the previous episode and was merged and called an episode span. ^d^ *n/a* signifies that there was *no* increase, at all, in vasopressor dose during the episode.

In Hospital 2, there were 26 episode spans. The majority of episode spans were not associated with an increase in vasopressor infusion dose (see Table 3). After reviewing the entirety of the medical record, we also observed that there was no documentation of any specific complication nor event at the time of any of these episodes (e.g., no arrhythmia, no gastrointestinal bleeding). For four of the episodes, there was a reduction of sedative infusion rate documented, and for another four episodes, there was a volume bolus (crystalloid, blood or albumen). The remainder lacked other documented clinical responses.

### Logistic regression model for predicting sustained hypotension

From the set of HR, MAP and vasopressor dose candidate features, there were no significant HR and vasopressor dose features. All the significant features were related to MAP (see Table 4). The associated logistic regression model was as follows:

1 $$\mathrm{log}[\frac{p}{1-p}]=-0.45{s}_{P}^{[5]}-4.19{m}_{P}^{[10]}+0.39{\sigma }_{P}^{[10]}+0.68{s}_{P}^{[45]}+0.22{\sigma }_{P}^{[60]}$$

where $p$ is the probability associated with the occurrence of a sustained hypotension 15 min forward in time, $m$, $s$, and $\sigma$ are the mean, least-squares slope, and standard deviation, the subscripts $P$ indicates MAP, and the superscript $[N]$ indicates the time scale ($N$ min) with which the features are computed.

**Table 4:** Mean arterial pressure (MAP) feature coefficients in the logistic regression model.

	MAP Mean	MAP Slope	MAP Std. Dev.
05 min	—	−0.45	—
10 min	−4.19	—	0.39
45 min	—	0.68	—
60 min	—	—	0.22

The β coefficients for significant features are listed; “-” indicates non-significant features. (None of the candidate features related to heart rate and vasopressor infusion dose were found to be statistically significant).

### Logistic regression model evaluation

For deciles of the training dataset, the model’s predicted versus the actual probabilities had a mean error of 0.0% and the 95% limits-of-agreement between the predicted versus actual incidences of forthcoming sustained hypotension were −3.4% to +3.4%, suggesting that the logistic regression model had adequate goodness-of-fit to the dataset.

**Figure 2:**
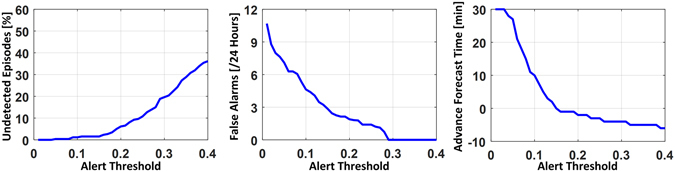
Relationship between diagnostic performance metrics versus alarm thresholds for the logistic regression model output. With increasing output threshold (i.e., a more stringent threshold for alarming), the model exhibited *i*) increasing rate of undetected episodes of hypotension; *ii*) decreasing false alarm rate; and *iii*) decreasing advance forecast time.

The trade-off between detection sensitivity and false alarms for different thresholds for the logistic regression model output are shown in Fig. 2. With increasing output threshold (i.e., a more stringent threshold for alarming), the model exhibited *i*) increasing likelihood of undetected episodes of hypotension; *ii*) decreasing false alarm rate; and *iii*) decreasing advance forecast time. The results of evaluating the logistic regression model in the Hospital 1 testing dataset are shown in Table 5. Overall, the logistic regression model provided advance warning that was significantly greater than MAP < 60 mmHg, and yielded significantly fewer false alarms than either of the simple threshold alerts (MAP < 60 mmHg and <65 mmHg). Figure 1 shows several examples of the model detecting an episode of hypotension, and also examples of false alarms.

**Table 5:** Performance of the logistic regression model versus two simple threshold alerts.

	Hospital 1			Hospital 2		
	Model	Threshold Alert		Model	Threshold Alert	
		60 mmHg	65 mmHg		60 mmHg	65 mmHg
**Statistics:**						
Number of sustained episodes of hypotension, n	**259**	**259**	**259**	**26**	**26**	**26**
Proportion of episodes that were undetected, %	**0.4%**	**0.0%**	**0.0%**	**0.0%**	**0.0%**	**0.0%**
Advance forecast time, median (IQR), min	**12.0** (0.0–30.0)	**0.0** ^†^ (0.0–0.0)	**5.0** (2.0–18.8)	**22.0** (2.0–30.0)	**0.0** ^†^ (0.0–0.0)	**8.0** (4.0–26.0)
Number of false alarms per 24 hours, median per stay (IQR)	**6.9** (2.0–12.4)	**11.9** ^*^ (4.2–20.9)	**25.9** ^†^ (12.6–42.6)	**2.1** (0.4–7.5)	**3.8** ^*^ (1.9–10.2)	**20.1** ^†^ (9.0–44.0)
Duration of false alarms, median per stay (IQR), min	**8.** **0** (3.0–16.0)	**2.0** ^†^ (1.0–3.0)	**3.0** ^†^ (1.0–7.0)	**5.** **0** (2.3–12.0)	**2.0** ^†^ (1.0–3.0)	**2.0** ^†^ (1.0–4.0)

Hypotension is defined as MAP < 60 mmHg and sustained episode of hypotension is defined as ≥15 min. An episode of hypotension was “detected” if, directly upon its onset, there had been an alarm episode in the preceding 30 min. An alarm episode (defined as the continuous time interval when the alarm condition was true) was a “false alarm” if, directly upon its onset, there was no sustained hypotension commencing within 30 min. *Significant difference between model and threshold alert, P < 0.05. ^†^Significant difference between model and threshold alert, P < 0.001.

Results from prospective evaluation in Hospital 2 are also shown in Table 5, where 62 ICU stays experienced 26 sustained episodes of hypotension. Performance in Hospital 2 was similar to performance in Hospital 1, in the sense that the logistic regression model again provided sensitive advance warning to episodes of hypotension, without a greater number of false alarms than the simple threshold alert. The model detected every episode of hypotension, and with a median advance forecast time of 22 min prior to the onset of the episode.

### Secondary analyses (analysis with relaxed data exclusion criteria and evaluation of additional candidate predictor features)

For the primary analysis, we excluded data intervals that made it hard to interpret the clinician dose-titration behaviors. In a secondary analysis, we repeated the evaluation of the logistic regression model, this time including data intervals from the initial 30 min of a patient’s ICU stay and during maximum vasopressor dosages and during infusion of two or more vasopressors at the same time. This did not alter our findings. Specifically, there were no statistically significant differences, as compared to the numerical results shown in Table 5. As well, our findings were the same: the logistic regression model again provided advance warning that was significantly greater than MAP < 60 mmHg, a non-significant trend toward greater advance warning than MAP < 65 mmHg, and the logistic regression model again yielded significantly fewer false alarms than either of the simple threshold alerts (MAP < 60 mmHg and <65 mmHg).

We found that gender and “presence of sepsis (yes/no)” were additional significant predictor features in the secondary logistic regression model. However, when this secondary model was tested and compared with the primary logistic regression model (i.e., model with only MAP-related predictor features), there was no improved performance. Specifically, there were no statistically significant differences, as compared to the numerical results shown in Table 5. Indeed, the secondary model had a non-significant trend towards worse (i.e., shorter) advance forecast time in both Hospital 1 and Hospital 2 datasets, and also a trend toward increased number of false alarms per 24 hours in Hospital 2 dataset.

### Comparing documented dose increases versus logistic regression model output

The logistic regression model was predicting sustained hypotension during 48% of the instances when the vasopressor dose was increased ≥ 50%. Within a wider 60 min window, the logistic regression model was predicting sustained hypotension during 76% of the instances.

## Discussion

There are three main findings of this analysis. First, ICU patients receiving vasopressor infusion commonly experienced sustained episodes of hypotension, with MAP below the limits of CNS auto-regulation often for 30 min or even longer. Classical physiological studies and retrospective analyses have suggested that such episodes cause deleterious hypoperfusion of the CNS and other end-organs. Second, most of these episodes developed in the absence of any preceding dose adjustments, and after commencing, many episodes persisted without any documented increases in vasopressor dose. This raises the possibility that many of the episodes were preventable via more vigilant clinical interventions, including vasopressor dose increases immediately upon the onset of the episodes, or dose increases before the onset (to prevent the episode of hypotension altogether). Third, a statistical model only requiring BP trend data as an input was able to predict the onset of these episodes well in advance of the onset, and with significantly fewer false alarms than simple threshold alerts. This suggests that advance warning prior to the onset of sustained hypotension is technically feasible.

The findings in this investigation arose from two medical centers, so while the generalizability of this finding is not known, it is evident that BP goals may not always be met during vasopressor infusions. A recent report about ICU patients with acute spinal cord injury (ASCI) revealed a similar failure to maintain MAP goals: although current ASCI guidelines advise maintenance of MAP of 85–90 mmHg for the first week after ASCI, the investigators reported that MAP was below that threshold for 42% of all documented values^10^.

The actual effect of these episodes of hypotension is not known, but there is ample indirect evidence that such hypotension is deleterious. In animal models it has been shown that the CNS cannot effectively autoregulate perfusion below MAP of 65 mmHg, which means that CNS ischemia is likely to occur^3, 4^. Therefore, low-level sustained hypotension might cause CNS ischemic injury. Indeed, major cognitive injury is a tragic sequelae in survivors of sepsis^11^ and it is reasonable to speculate that sustained hypotension could be directly causing some of the cognitive injury. Clinical studies have shown a correlation between hypotension and worse ICU outcomes^5–7^ but of course in retrospective studies, it is not possible to distinguish between causal versus correlative relationships.

Overall, further study of the actual consequences of sustained low-level hypotension on neurons and nephrons may be advisable, to establish the true risk of sustained hypotension during ICU care. If a causal relationship between hypotension and patient injury is established, it would behoove clinical guidelines to strengthen their guidance (conversely, if sustained hypotension proves to be well-tolerated, the guidelines should be relaxed).

Are sustained episodes of hypotension during vasopressor therapy in the ICU preventable? We found that most episodes of hypotension occurred not because the vasopressor had been excessively weaned in the time preceding the episode, nor because there had been an insufficient increase in vasopressor dose preceding the episode. Rather, most episodes of hypotension occurred in the absence of any preceding vasopressor dose change. As well, when we reviewed the nursing notes from the time of the episodes, we also did not find any other clinical events documented to explain the episode. In other words, in none of the cases that we evaluated did the patient experience sudden-onset problem such as new onset GI bleeding or new arrhythmias. Typically, for our sustained episodes of hypotension, the MAP simply drifted out of goal range.

Moreover, the clinical response to sustained episodes of hypotension often lacked any vasopressor dose increase, nor consistent responses such as volume boluses. In Hospital 1, the top quartile of sustained episodes of hypotension persisted longer than 72 min (see Table 3) without an associated vasopressor dose increase. It is notable that, for the minority of episodes in which the vasopressor dose was indeed increased in Hospital 1, a reduced duration of hypotension was observed.

Taken together, these findings support the notion that the clinicians often failed to initiate interventions that could have prevented or shortened these episodes. A passive response to hypotension may reflect some aspect of clinical inertia^14^, a well-known phenomenon in which clinicians err by delaying an appropriate intervention. We speculate that one factor that may promote clinical inertia during episodes of hypotension is that hypotension can be clinically indistinct: aside from the low MAP displayed on a monitor, there may not be other direct abnormalities, i.e., no observable cyanosis, posturing, nor abnormal respirations.

In general, we found a frequent failure to document any intervention before or during episodes of hypotension, and it is reasonable to speculate that many of these episodes might have been prevented with proactive interventions. On the assumption that sustained hypotension is deleterious, it may be prudent for intensivists to conduct quality-assurance review within their local ICUs to ensure MAP is being adequately maintained and to conduct in-service training to encourage staff to avoid clinical inertia and adhere to BP goals.

Although the prediction of hypotension in an ICU (e.g. early warning systems) has been explored in other reports^15, 16^, we believe that ours is the first to explicitly focus on the development of sustained hypotension during ongoing vasopressor infusion. The goal was to develop an informatics tool for alerting clinicians that a vasopressor dose increase (or other intervention) may be clinically indicated.

Our analysis suggests that advance warning is quite feasible based solely on statistical associations between BP trend data and subsequent hypotension: a relatively simple logistic regression model was able to predict episodes of hypotension well before their onset. Performance was favorable both in the testing dataset from Hospital 1, and also in the dataset collected prospectively from Hospital 2. Specifically, the logistic regression model was able to detect almost all of the sustained episodes of hypotension with advance warning, and with fewer false alarms, relative to a simple threshold alert (see Table 5).

Is it surprising that a logistic regression model relying only on features related to MAP trends performed this well? Perhaps not, considering that we found that most episodes of hypotension involved MAP slowly drifting out of range (e.g., Fig. 1). Indeed, the significant MAP features were intuitive: lower MAP values, negative MAP slopes, and high MAP standard deviation (i.e., variability) all raised the likelihood of a future episode of hypotension.

It may be surprising that HR was not found to be a significant independent parameter during model development in the Hospital 1 training dataset. However, even without HR, the model performed well in the prospective Hospital 2 dataset, suggesting that the model was valid.

We also observed that vasopressor dose was not a significant parameter during model development. We believe that infrequent vasopressor dose changes was the primary reason why it was not useful for the model; indeed, noting that the median time between dose changes was found to be >60 min (which is the length of the feature extraction window), vasopressor dose was essentially constant in a median patient in the feature extraction window. Evidently, the absolute value of the dose was not strongly associated with the likelihood of an imminent episode of hypotension.

The fact that the statistical model only required MAP input data is a strength for real-world implementation, since electronic MAP data are accessible via serial port interface to most vital-sign monitors. By contrast, a predictive model that requires additional clinical data would likely require electronic communication with the patient’s electronic medical record (EMR), which can be challenging (EMR providers can be resistant to interoperability for reasons of data security, liability concern, and business strategy, and also, different medical centers organize EMR data using different database structures using different concept semantics)^17, 18^.

“False alarms” by the logistic regression model occurred when the MAP was trending towards hypotension but never actually developed sustained hypotension (see Fig. 1). Under such circumstances, clinical alerting may be justifiable. Accordingly, the overall alarm characteristics of the logistic regression model may be reasonable assuming that advance warning can help prevent deleterious hypotension (see subsection below).

To be sure, predictive performance may further improve with additional clinical data, or with more sophisticated statistical methods. Logistic regression is a standard, simple classification technique, and we found that the logistic regression model offered consistent performance between Hospital 1 retrospective testing and Hospital 2 prospective testing (see Table 5), which supports the model’s applicability and generalizability, even if the linearity assumption of the logistic regression model was not strictly applicable to this analysis. In future work, more sophisticated methods, using regularization techniques or alternative classifiers, as well as additional clinical input data, might indeed perform even better. As a practical matter, the logistic regression model’s false alarm rate might be the focus for further improvement, since the model already offered highly sensitive advance warning.

Finally, we consider the actual clinical value of a statistical model to provide advance warning prior to the onset of sustained hypotension: to prompt clinicians to intervene and either prevent the episode of hypotension altogether, or at least be prepared to act quickly upon its onset. Assuming that sustained episodes of hypotension are deleterious to the CNS or other vital organs, it would be suitable to prevent the episode with increases in vasopressor dose.

Of course, advance warning is not strictly necessary. A simple threshold alert would offer many of the same clinical benefits, assuming that clinicians quickly responded when patients developed hypotension. Alternatively, it may be that ICUs need to adopt superior “dosing policies” that promote dose titration to prevent hypotension. Therefore, advance warning relying on an automated statistical model is not the only potential solution to improved compliance with BP goals during vasopressor therapy.

What is established from our results is the feasibility of a statistical model to provide advance warning of hypotension. As noted above, this model also had the practical advantage that it only requires an interface with the patient’s bedside monitor. Therefore, while there may also be other alternatives to consider, this model may offer a promising tool that could promote adherence with BP goals during vasopressor therapy.

There are several limitations to this report. First, regarding the incidence of sustained hypotension during vasopressor therapy, our findings were only from two hospitals. The degree to which this problem is widespread is not known, although it has been corroborated by another study of preventable hypotension in an ICU^10^. Second, whether sustained hypotension below the commonly accepted threshold of CNS auto-regulation is truly deleterious is not known. It may be inconsequential, or it may be a substantial contributor to mortality and morbidity such as the well-known cognitive sequelae sepsis^11^. Third, although the logistic regression model offers promising test characteristics for advance warning of imminent sustained hypotension, whether or not giving clinicians advance warning would truly lead to tighter adherence to BP goals is speculative and requires clinical investigation. Also, it would be important to employ the logistic regression model only as a safety measure and not as a primary means of determining when dose changes are necessary, since half of all documented dose changes did not occur at an instance when the logistic regression model predicted sustained hypotension. Finally, there may be superior alternatives to the model investigated in this report using other clinical inputs or statistical methods. In particular, vasopressor dosage may be a more predictive feature if more precise data were investigated (rather than the dose documented in the nursing flowcharts).

In conclusion, during vasopressor therapy, sustained episodes of hypotension were common and typically without associated vasopressor dose increases. Because the likelihood is that sustained hypotension causes CNS and other end-organ injury, the prevalence of this practice and its clinical consequences should be further investigated. In a prospective validation, a logistic regression model demonstrated promising test characteristics and it may offer one tool for reducing hypotension during vasopressor therapy in the ICU.
